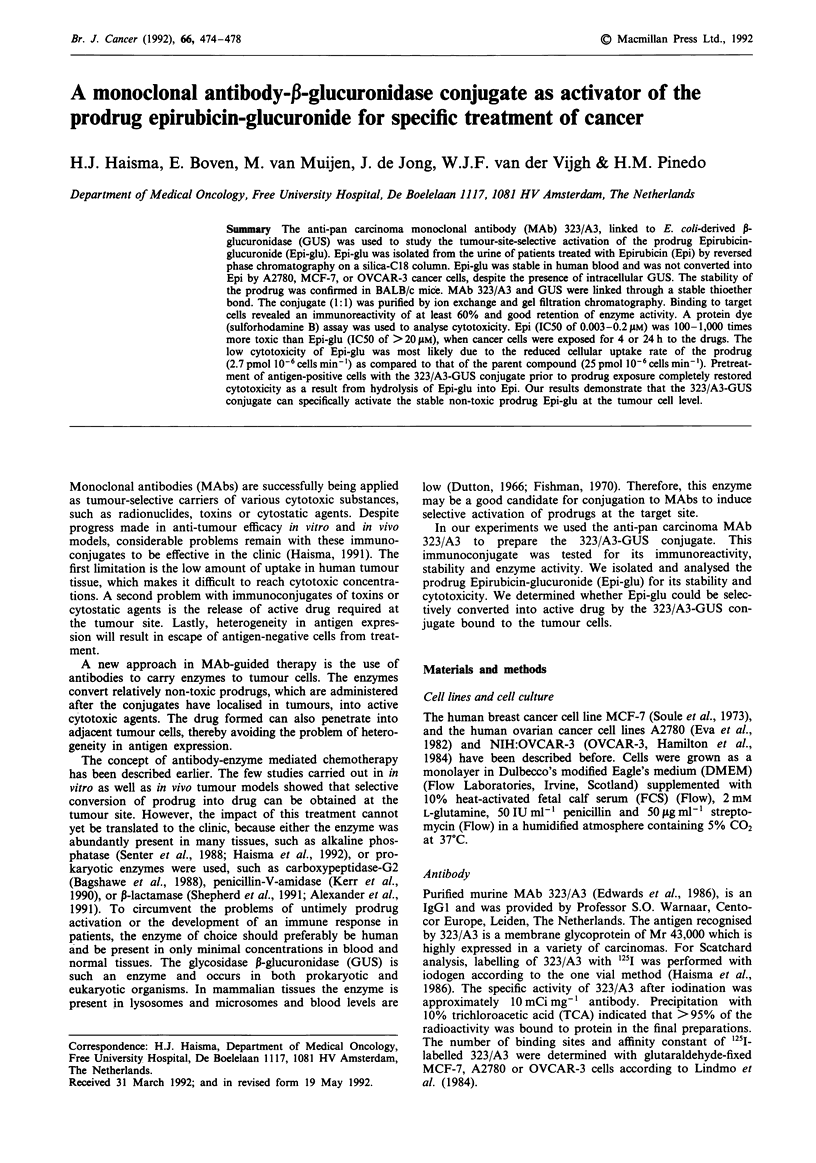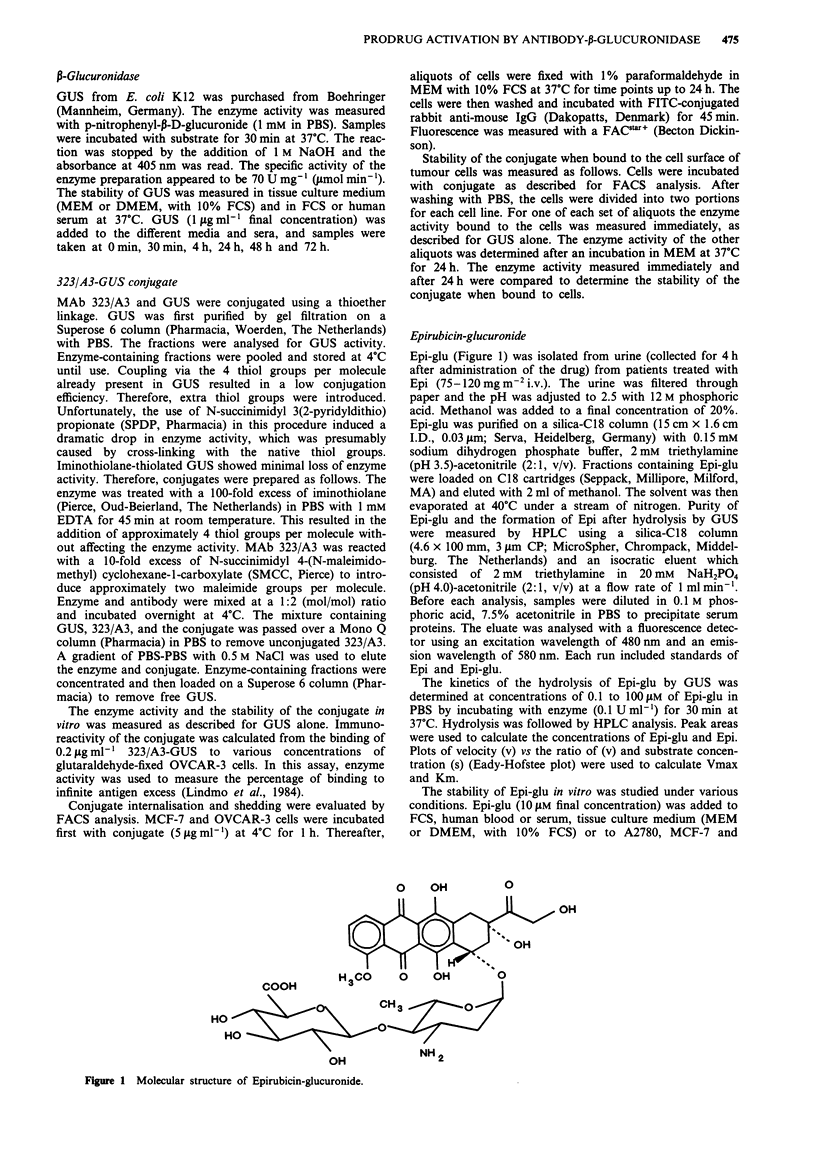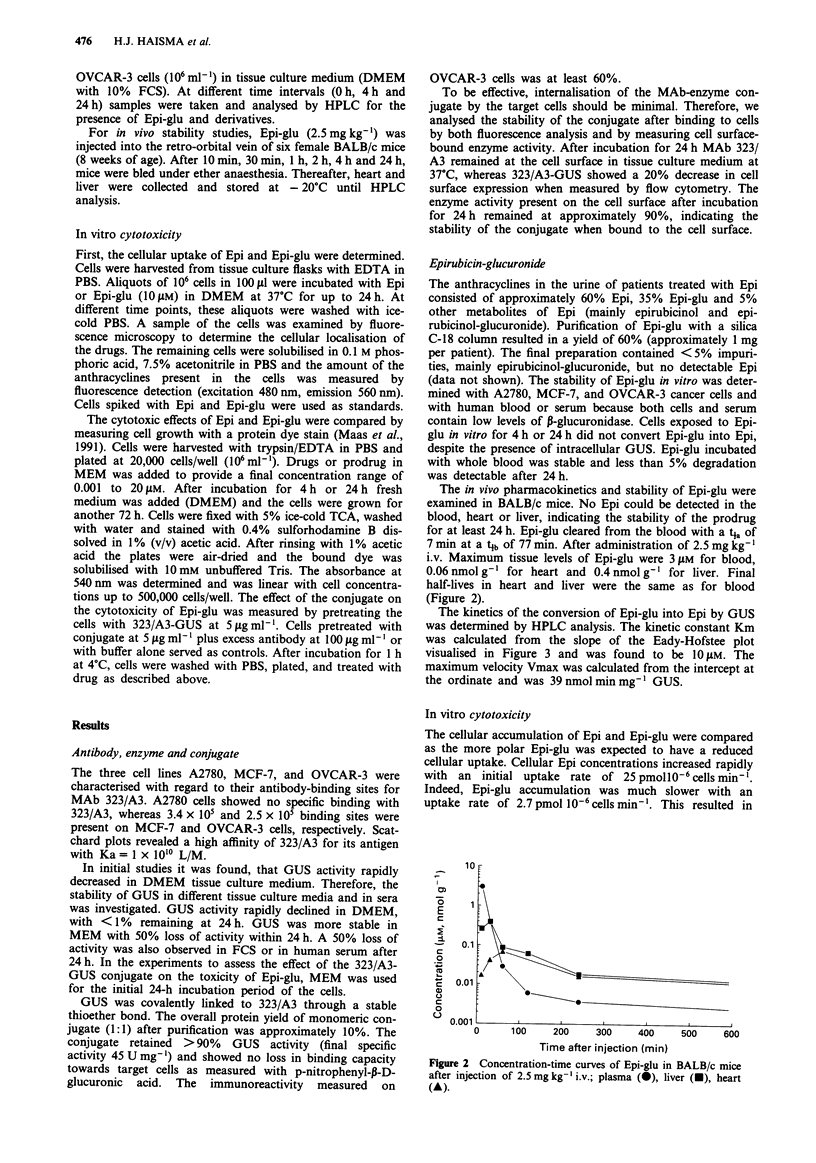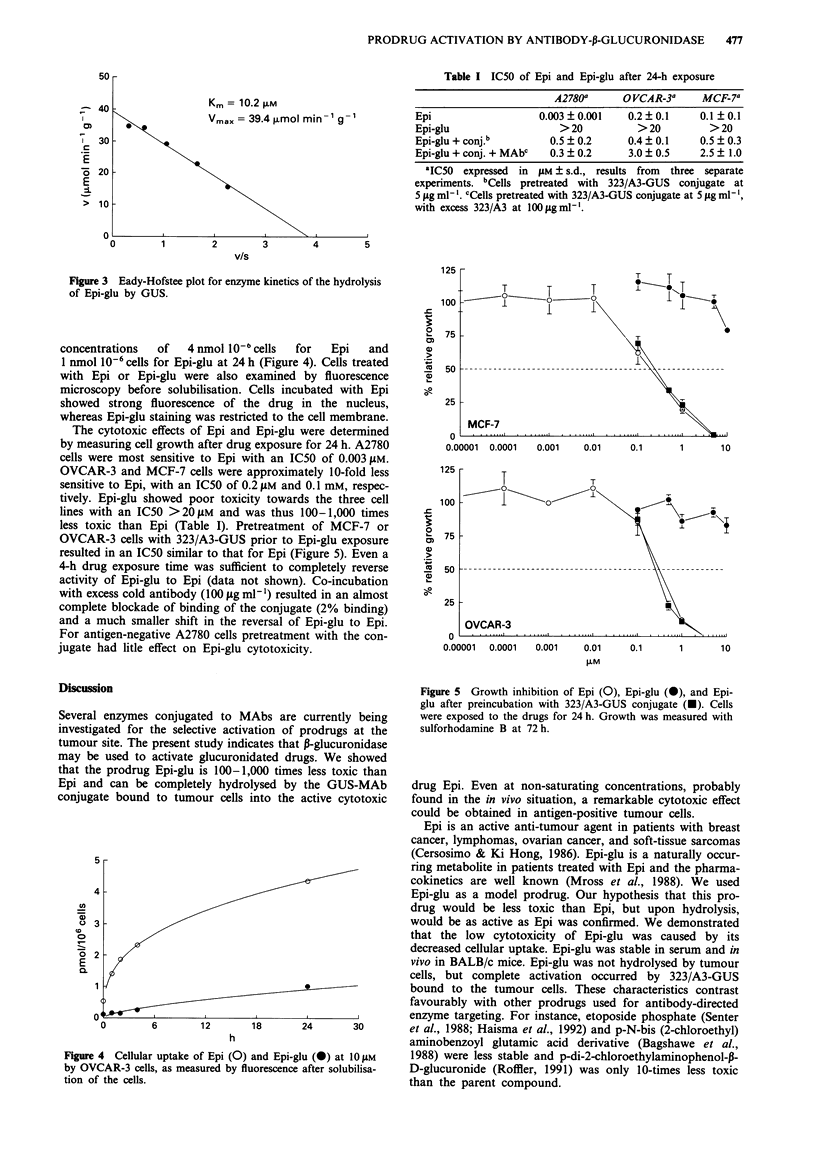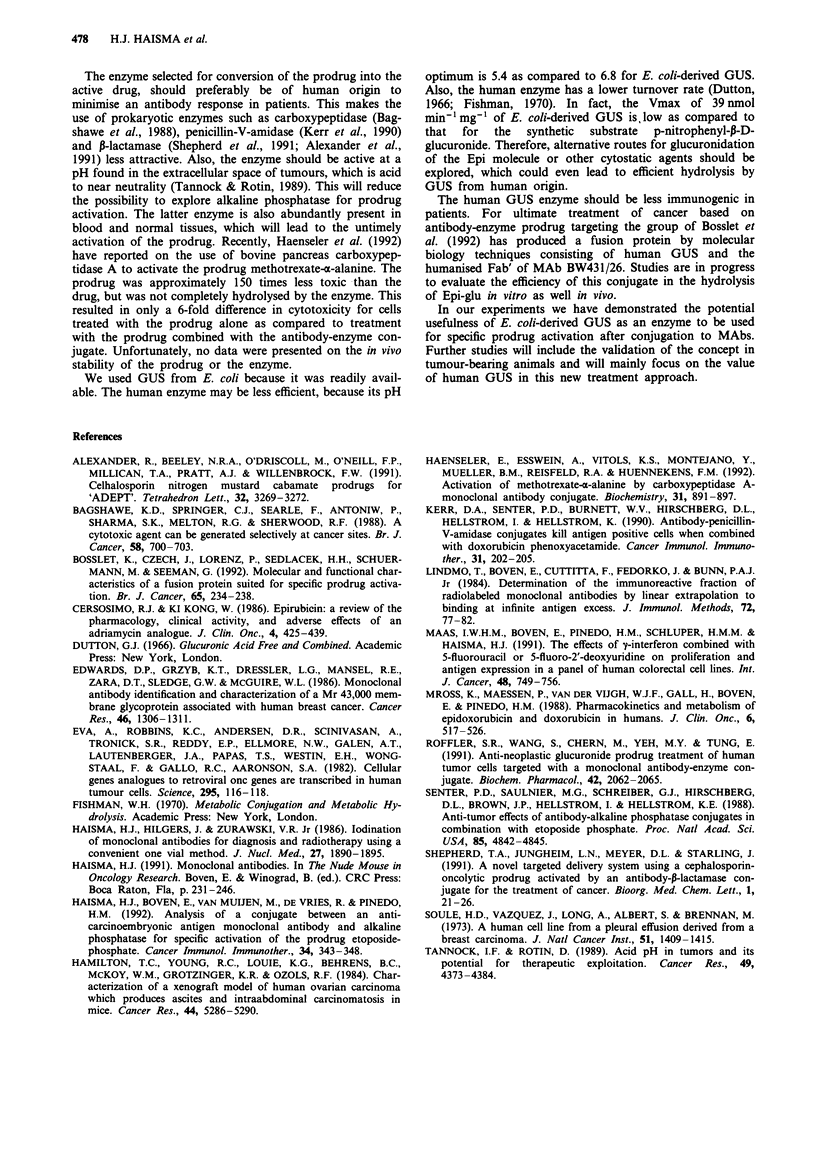# A monoclonal antibody-beta-glucuronidase conjugate as activator of the prodrug epirubicin-glucuronide for specific treatment of cancer.

**DOI:** 10.1038/bjc.1992.298

**Published:** 1992-09

**Authors:** H. J. Haisma, E. Boven, M. van Muijen, J. de Jong, W. J. van der Vijgh, H. M. Pinedo

**Affiliations:** Department of Medical Oncology, Free University Hospital, Amsterdam, The Netherlands.

## Abstract

The anti-pan carcinoma monoclonal antibody (MAb) 323/A3, linked to E. coli-derived beta-glucuronidase (GUS) was used to study the tumour-site-selective activation of the prodrug Epirubicin-glucuronide (Epi-glu). Epi-glu was isolated from the urine of patients treated with Epirubicin (Epi) by reversed phase chromatography on a silica-C18 column. Epi-glu was stable in human blood and was not converted into Epi by A2780, MCF-7, or OVCAR-3 cancer cells, despite the presence of intracellular GUS. The stability of the prodrug was confirmed in BALB/c mice. MAb 323/A3 and GUS were linked through a stable thioether bond. The conjugate (1:1) was purified by ion exchange and gel filtration chromatography. Binding to target cells revealed an immunoreactivity of at least 60% and good retention of enzyme activity. A protein dye (sulforhodamine B) assay was used to analyse cytotoxicity. Epi (IC50 of 0.003-0.2 microM) was 100-1,000 times more toxic than Epi-glu (IC50 of greater than 20 microM), when cancer cells were exposed for 4 or 24 h to the drugs. The low cytotoxicity of Epi-glu was most likely due to the reduced cellular uptake rate of the prodrug (2.7 pmol 10(-6) cells min-1) as compared to that of the parent compound (25 pmol 10(-6) cells min-1). Pretreatment of antigen-positive cells with the 323/A3-GUS conjugate prior to prodrug exposure completely restored cytotoxicity as a result from hydrolysis of Epi-glu into Epi. Our results demonstrate that the 323/A3-GUS conjugate can specifically activate the stable non-toxic prodrug Epi-glu at the tumour cell level.


Br. J. Cancer (1992), 66, 474–478 © Macmillan Press Ltd., 1992

### A monoclonal antibody-β-glucuronidase conjugate as activator of the prodrug epirubicin-glucuronide for specific treatment of cancer

H.J. Haisma, E. Boven, M. van Muijen, J. de Jong, W.J.F. van der Vijgh & H.M. Pinedo

*Department of Medical Oncology, Free University Hospital, De Boelelaan 1117, 1081 HV Amsterdam, The Netherlands*

**Summary** The anti-pan carcinoma monoclonal antibody (MAb) 323/A3, linked to *E. coli*-derived β-glucuronidase (GUS) was used to study the tumour-site-selective activation of the prodrug Epirubicin-glucuronide (Epi-glu). Epi-glu was isolated from the urine of patients treated with Epirubicin (Epi) by reversed phase chromatography on a silica-C18 column. Epi-glu was stable in human blood and was not converted into Epi by A2780, MCF-7, or OVCAR-3 cancer cells, despite the presence of intracellular GUS. The stability of the prodrug was confirmed in BALB/c mice. MAb 323/A3 and GUS were linked through a stable thioether bond. The conjugate (1:1) was purified by ion exchange and gel filtration chromatography. Binding to target cells revealed an immunoreactivity of at least 60% and good retention of enzyme activity. A protein dye (sulforhodamine B) assay was used to analyse cytotoxicity. Epi (IC50 of 0.003–0.2 μM) was 100–1,000 times more toxic than Epi-glu (IC50 of >20 μM), when cancer cells were exposed for 4 or 24 h to the drugs. The low cytotoxicity of Epi-glu was most likely due to the reduced cellular uptake rate of the prodrug (2.7 pmol $10^{-6}$ cells $min^{-1}$) as compared to that of the parent compound (25 pmol $10^{-6}$ cells $min^{-1}$). Pretreatment of antigen-positive cells with the 323/A3-GUS conjugate prior to prodrug exposure completely restored cytotoxicity as a result from hydrolysis of Epi-glu into Epi. Our results demonstrate that the 323/A3-GUS conjugate can specifically activate the stable non-toxic prodrug Epi-glu at the tumour cell level.

Monoclonal antibodies (MAbs) are successfully being applied as tumour-selective carriers of various cytotoxic substances, such as radionuclides, toxins or cytostatic agents. Despite progress made in anti-tumour efficacy *in vitro* and *in vivo* models, considerable problems remain with these immunoconjugates to be effective in the clinic (Haisma, 1991). The first limitation is the low amount of uptake in human tumour tissue, which makes it difficult to reach cytotoxic concentrations. A second problem with immunoconjugates of toxins or cytostatic agents is the release of active drug required at the tumour site. Lastly, heterogeneity in antigen expression will result in escape of antigen-negative cells from treatment.

A new approach in MAb-guided therapy is the use of antibodies to carry enzymes to tumour cells. The enzymes convert relatively non-toxic prodrugs, which are administered after the conjugates have localised in tumours, into active cytotoxic agents. The drug formed can also penetrate into adjacent tumour cells, thereby avoiding the problem of heterogeneity in antigen expression.

The concept of antibody-enzyme mediated chemotherapy has been described earlier. The few studies carried out in *in vitro* as well as *in vivo* tumour models showed that selective conversion of prodrug into drug can be obtained at the tumour site. However, the impact of this treatment cannot yet be translated to the clinic, because either the enzyme was abundantly present in many tissues, such as alkaline phosphatase (Senter *et al.*, 1988; Haisma *et al.*, 1992), or prokaryotic enzymes were used, such as carboxypeptidase-G2 (Bagshawe *et al.*, 1988), penicillin-V-amidase (Kerr *et al.*, 1990), or β-lactamase (Shepherd *et al.*, 1991; Alexander *et al.*, 1991). To circumvent the problems of untimely prodrug activation or the development of an immune response in patients, the enzyme of choice should preferably be human and be present in only minimal concentrations in blood and normal tissues. The glycosidase β-glucuronidase (GUS) is such an enzyme and occurs in both prokaryotic and eukaryotic organisms. In mammalian tissues the enzyme is present in lysosomes and microsomes and blood levels are low (Dutton, 1966; Fishman, 1970). Therefore, this enzyme may be a good candidate for conjugation to MAbs to induce selective activation of prodrugs at the target site.

In our experiments we used the anti-pan carcinoma MAb 323/A3 to prepare the 323/A3-GUS conjugate. This immunoconjugate was tested for its immunoreactivity, stability and enzyme activity. We isolated and analysed the prodrug Epirubicin-glucuronide (Epi-glu) for its stability and cytotoxicity. We determined whether Epi-glu could be selectively converted into active drug by the 323/A3-GUS conjugate bound to the tumour cells.

Correspondence: H.J. Haisma, Department of Medical Oncology, Free University Hospital, De Boelelaan 1117, 1081 HV Amsterdam, The Netherlands.
Received 31 March 1992; and in revised form 19 May 1992.

### Materials and methods

### Cell lines and cell culture

The human breast cancer cell line MCF-7 (Soule *et al.*, 1973), and the human ovarian cancer cell lines A2780 (Eva *et al.*, 1982) and NIH:OVCAR-3 (OVCAR-3, Hamilton *et al.*, 1984) have been described before. Cells were grown as a monolayer in Dulbecco's modified Eagle's medium (DMEM) (Flow Laboratories, Irvine, Scotland) supplemented with 10% heat-activated fetal calf serum (FCS) (Flow), 2 mM L-glutamine, 50 IU $ml^{-1}$ penicillin and 50 μg $ml^{-1}$ streptomycin (Flow) in a humidified atmosphere containing 5% $CO_2$ at 37°C.

### Antibody

Purified murine MAb 323/A3 (Edwards *et al.*, 1986), is an IgG1 and was provided by Professor S.O. Warnaar, Centocor Europe, Leiden, The Netherlands. The antigen recognised by 323/A3 is a membrane glycoprotein of Mr 43,000 which is highly expressed in a variety of carcinomas. For Scatchard analysis, labelling of 323/A3 with $^{125}I$ was performed with iodogen according to the one vial method (Haisma *et al.*, 1986). The specific activity of 323/A3 after iodination was approximately 10 mCi $mg^{-1}$ antibody. Precipitation with 10% trichloroacetic acid (TCA) indicated that >95% of the radioactivity was bound to protein in the final preparations. The number of binding sites and affinity constant of $^{125}I$-labelled 323/A3 were determined with glutaraldehyde-fixed MCF-7, A2780 or OVCAR-3 cells according to Lindmo *et al.* (1984).

### *β-Glucuronidase*

GUS from *E. coli* K12 was purchased from Boehringer (Mannheim, Germany). The enzyme activity was measured with p-nitrophenyl-β-D-glucuronide (1 mM in PBS). Samples were incubated with substrate for 30 min at 37°C. The reaction was stopped by the addition of 1 M NaOH and the absorbance at 405 nm was read. The specific activity of the enzyme preparation appeared to be 70 U $mg^{-1}$ (μmol $min^{-1}$). The stability of GUS was measured in tissue culture medium (MEM or DMEM, with 10% FCS) and in FCS or human serum at 37°C. GUS (1 μg $ml^{-1}$ final concentration) was added to the different media and sera, and samples were taken at 0 min, 30 min, 4 h, 24 h, 48 h and 72 h.

### *323/A3-GUS conjugate*

MAb 323/A3 and GUS were conjugated using a thioether linkage. GUS was first purified by gel filtration on a Superose 6 column (Pharmacia, Woerden, The Netherlands) with PBS. The fractions were analysed for GUS activity. Enzyme-containing fractions were pooled and stored at 4°C until use. Coupling via the 4 thiol groups per molecule already present in GUS resulted in a low conjugation efficiency. Therefore, extra thiol groups were introduced. Unfortunately, the use of N-succinimidyl 3(2-pyridyldithio) propionate (SPDP, Pharmacia) in this procedure induced a dramatic drop in enzyme activity, which was presumably caused by cross-linking with the native thiol groups. Iminothiolane-thiolated GUS showed minimal loss of enzyme activity. Therefore, conjugates were prepared as follows. The enzyme was treated with a 100-fold excess of iminothiolane (Pierce, Oud-Beierland, The Netherlands) in PBS with 1 mM EDTA for 45 min at room temperature. This resulted in the addition of approximately 4 thiol groups per molecule without affecting the enzyme activity. MAb 323/A3 was reacted with a 10-fold excess of N-succinimidyl 4-(N-maleimidomethyl) cyclohexane-1-carboxylate (SMCC, Pierce) to introduce approximately two maleimide groups per molecule. Enzyme and antibody were mixed at a 1:2 (mol/mol) ratio and incubated overnight at 4°C. The mixture containing GUS, 323/A3, and the conjugate was passed over a Mono Q column (Pharmacia) in PBS to remove unconjugated 323/A3. A gradient of PBS-PBS with 0.5 M NaCl was used to elute the enzyme and conjugate. Enzyme-containing fractions were concentrated and then loaded on a Superose 6 column (Pharmacia) to remove free GUS.

The enzyme activity and the stability of the conjugate *in vitro* was measured as described for GUS alone. Immunoreactivity of the conjugate was calculated from the binding of 0.2 μg $ml^{-1}$ 323/A3-GUS to various concentrations of glutaraldehyde-fixed OVCAR-3 cells. In this assay, enzyme activity was used to measure the percentage of binding to infinite antigen excess (Lindmo *et al.*, 1984).

Conjugate internalisation and shedding were evaluated by FACS analysis. MCF-7 and OVCAR-3 cells were incubated first with conjugate (5 μg $ml^{-1}$) at 4°C for 1 h. Thereafter, aliquots of cells were fixed with 1% paraformaldehyde in MEM with 10% FCS at 37°C for time points up to 24 h. The cells were then washed and incubated with FITC-conjugated rabbit anti-mouse IgG (Dakopatts, Denmark) for 45 min. Fluorescence was measured with a $FAC^{star+}$ (Becton Dickinson).

Stability of the conjugate when bound to the cell surface of tumour cells was measured as follows. Cells were incubated with conjugate as described for FACS analysis. After washing with PBS, the cells were divided into two portions for each cell line. For one of each set of aliquots the enzyme activity bound to the cells was measured immediately, as described for GUS alone. The enzyme activity of the other aliquots was determined after an incubation in MEM at 37°C for 24 h. The enzyme activity measured immediately and after 24 h were compared to determine the stability of the conjugate when bound to cells.

### *Epirubicin-glucuronide*

Epi-glu (Figure 1) was isolated from urine (collected for 4 h after administration of the drug) from patients treated with Epi (75–120 mg $m^{-2}$ i.v.). The urine was filtered through paper and the pH was adjusted to 2.5 with 12 M phosphoric acid. Methanol was added to a final concentration of 20%. Epi-glu was purified on a silica-C18 column (15 cm × 1.6 cm I.D., 0.03 μm; Serva, Heidelberg, Germany) with 0.15 mM sodium dihydrogen phosphate buffer, 2 mM triethylamine (pH 3.5)-acetonitrile (2:1, v/v). Fractions containing Epi-glu were loaded on C18 cartridges (Seppack, Millipore, Milford, MA) and eluted with 2 ml of methanol. The solvent was then evaporated at 40°C under a stream of nitrogen. Purity of Epi-glu and the formation of Epi after hydrolysis by GUS were measured by HPLC using a silica-C18 column (4.6 × 100 mm, 3 μm CP; MicroSpher, Chrompack, Middelburg. The Netherlands) and an isocratic eluent which consisted of 2 mM triethylamine in 20 mM $NaH_2PO_4$ (pH 4.0)-acetonitrile (2:1, v/v) at a flow rate of 1 ml $min^{-1}$. Before each analysis, samples were diluted in 0.1 M phosphoric acid, 7.5% acetonitrile in PBS to precipitate serum proteins. The eluate was analysed with a fluorescence detector using an excitation wavelength of 480 nm and an emission wavelength of 580 nm. Each run included standards of Epi and Epi-glu.

The kinetics of the hydrolysis of Epi-glu by GUS was determined at concentrations of 0.1 to 100 μM of Epi-glu in PBS by incubating with enzyme (0.1 U $ml^{-1}$) for 30 min at 37°C. Hydrolysis was followed by HPLC analysis. Peak areas were used to calculate the concentrations of Epi-glu and Epi. Plots of velocity (v) *vs* the ratio of (v) and substrate concentration (s) (Eady-Hofstee plot) were used to calculate Vmax and Km.

The stability of Epi-glu *in vitro* was studied under various conditions. Epi-glu (10 μM final concentration) was added to FCS, human blood or serum, tissue culture medium (MEM or DMEM, with 10% FCS) or to A2780, MCF-7 and

**Figure 1** Molecular structure of Epirubicin-glucuronide.

OVCAR-3 cells ($10^6$ ml$^{-1}$) in tissue culture medium (DMEM with 10% FCS). At different time intervals (0 h, 4 h and 24 h) samples were taken and analysed by HPLC for the presence of Epi-glu and derivatives.

For *in vivo* stability studies, Epi-glu (2.5 mg kg$^{-1}$) was injected into the retro-orbital vein of six female BALB/c mice (8 weeks of age). After 10 min, 30 min, 1 h, 2 h, 4 h and 24 h, mice were bled under ether anaesthesia. Thereafter, heart and liver were collected and stored at − 20°C until HPLC analysis.

### In vitro *cytotoxicity*

First, the cellular uptake of Epi and Epi-glu were determined. Cells were harvested from tissue culture flasks with EDTA in PBS. Aliquots of $10^6$ cells in 100 µl were incubated with Epi or Epi-glu (10 µM) in DMEM at 37°C for up to 24 h. At different time points, these aliquots were washed with ice-cold PBS. A sample of the cells was examined by fluorescence microscopy to determine the cellular localisation of the drugs. The remaining cells were solubilised in 0.1 M phosphoric acid, 7.5% acetonitrile in PBS and the amount of the anthracyclines present in the cells was measured by fluorescence detection (excitation 480 nm, emission 560 nm). Cells spiked with Epi and Epi-glu were used as standards.

The cytotoxic effects of Epi and Epi-glu were compared by measuring cell growth with a protein dye stain (Maas *et al.*, 1991). Cells were harvested with trypsin/EDTA in PBS and plated at 20,000 cells/well ($10^6$ ml$^{-1}$). Drugs or prodrug in MEM was added to provide a final concentration range of 0.001 to 20 µM. After incubation for 4 h or 24 h fresh medium was added (DMEM) and the cells were grown for another 72 h. Cells were fixed with 5% ice-cold TCA, washed with water and stained with 0.4% sulforhodamine B dissolved in 1% (v/v) acetic acid. After rinsing with 1% acetic acid the plates were air-dried and the bound dye was solubilised with 10 mM unbuffered Tris. The absorbance at 540 nm was determined and was linear with cell concentrations up to 500,000 cells/well. The effect of the conjugate on the cytotoxicity of Epi-glu was measured by pretreating the cells with 323/A3-GUS at 5 µg ml$^{-1}$. Cells pretreated with conjugate at 5 µg ml$^{-1}$ plus excess antibody at 100 µg ml$^{-1}$ or with buffer alone served as controls. After incubation for 1 h at 4°C, cells were washed with PBS, plated, and treated with drug as described above.

### Results

### *Antibody, enzyme and conjugate*

The three cell lines A2780, MCF-7, and OVCAR-3 were characterised with regard to their antibody-binding sites for MAb 323/A3. A2780 cells showed no specific binding with 323/A3, whereas $3.4 \times 10^5$ and $2.5 \times 10^5$ binding sites were present on MCF-7 and OVCAR-3 cells, respectively. Scatchard plots revealed a high affinity of 323/A3 for its antigen with Ka = $1 \times 10^{10}$ L/M.

In initial studies it was found, that GUS activity rapidly decreased in DMEM tissue culture medium. Therefore, the stability of GUS in different tissue culture media and in sera was investigated. GUS activity rapidly declined in DMEM, with <1% remaining at 24 h. GUS was more stable in MEM with 50% loss of activity within 24 h. A 50% loss of activity was also observed in FCS or in human serum after 24 h. In the experiments to assess the effect of the 323/A3-GUS conjugate on the toxicity of Epi-glu, MEM was used for the initial 24-h incubation period of the cells.

GUS was covalently linked to 323/A3 through a stable thioether bond. The overall protein yield of monomeric conjugate (1:1) after purification was approximately 10%. The conjugate retained >90% GUS activity (final specific activity 45 U mg$^{-1}$) and showed no loss in binding capacity towards target cells as measured with p-nitrophenyl-β-D-glucuronic acid. The immunoreactivity measured on OVCAR-3 cells was at least 60%.

To be effective, internalisation of the MAb-enzyme conjugate by the target cells should be minimal. Therefore, we analysed the stability of the conjugate after binding to cells by both fluorescence analysis and by measuring cell surface-bound enzyme activity. After incubation for 24 h MAb 323/A3 remained at the cell surface in tissue culture medium at 37°C, whereas 323/A3-GUS showed a 20% decrease in cell surface expression when measured by flow cytometry. The enzyme activity present on the cell surface after incubation for 24 h remained at approximately 90%, indicating the stability of the conjugate when bound to the cell surface.

### *Epirubicin-glucuronide*

The anthracyclines in the urine of patients treated with Epi consisted of approximately 60% Epi, 35% Epi-glu and 5% other metabolites of Epi (mainly epirubicinol and epirubicinol-glucuronide). Purification of Epi-glu with a silica C-18 column resulted in a yield of 60% (approximately 1 mg per patient). The final preparation contained <5% impurities, mainly epirubicinol-glucuronide, but no detectable Epi (data not shown). The stability of Epi-glu *in vitro* was determined with A2780, MCF-7, and OVCAR-3 cancer cells and with human blood or serum because both cells and serum contain low levels of β-glucuronidase. Cells exposed to Epi-glu *in vitro* for 4 h or 24 h did not convert Epi-glu into Epi, despite the presence of intracellular GUS. Epi-glu incubated with whole blood was stable and less than 5% degradation was detectable after 24 h.

The *in vivo* pharmacokinetics and stability of Epi-glu were examined in BALB/c mice. No Epi could be detected in the blood, heart or liver, indicating the stability of the prodrug for at least 24 h. Epi-glu cleared from the blood with a $t_{1/2\alpha}$ of 7 min at a $t_{1/2\beta}$ of 77 min. After administration of 2.5 mg kg$^{-1}$ i.v. Maximum tissue levels of Epi-glu were 3 µM for blood, 0.06 nmol g$^{-1}$ for heart and 0.4 nmol g$^{-1}$ for liver. Final half-lives in heart and liver were the same as for blood (Figure 2).

The kinetics of the conversion of Epi-glu into Epi by GUS was determined by HPLC analysis. The kinetic constant Km was calculated from the slope of the Eady-Hofstee plot visualised in Figure 3 and was found to be 10 µM. The maximum velocity Vmax was calculated from the intercept at the ordinate and was 39 nmol min mg$^{-1}$ GUS.

### In vitro *cytotoxicity*

The cellular accumulation of Epi and Epi-glu were compared as the more polar Epi-glu was expected to have a reduced cellular uptake. Cellular Epi concentrations increased rapidly with an initial uptake rate of 25 pmol$10^{-6}$ cells min$^{-1}$. Indeed, Epi-glu accumulation was much slower with an uptake rate of 2.7 pmol $10^{-6}$ cells min$^{-1}$. This resulted in

**Figure 2** Concentration-time curves of Epi-glu in BALB/c mice after injection of 2.5 mg kg$^{-1}$ i.v.; plasma (●), liver (■), heart (▲).

**Figure 3** Eady-Hofstee plot for enzyme kinetics of the hydrolysis of Epi-glu by GUS.

concentrations of 4 nmol $10^{-6}$ cells for Epi and 1 nmol $10^{-6}$ cells for Epi-glu at 24 h (Figure 4). Cells treated with Epi or Epi-glu were also examined by fluorescence microscopy before solubilisation. Cells incubated with Epi showed strong fluorescence of the drug in the nucleus, whereas Epi-glu staining was restricted to the cell membrane.

The cytotoxic effects of Epi and Epi-glu were determined by measuring cell growth after drug exposure for 24 h. A2780 cells were most sensitive to Epi with an IC50 of 0.003 μM. OVCAR-3 and MCF-7 cells were approximately 10-fold less sensitive to Epi, with an IC50 of 0.2 μM and 0.1 mM, respectively. Epi-glu showed poor toxicity towards the three cell lines with an IC50 >20 μM and was thus 100–1,000 times less toxic than Epi (Table I). Pretreatment of MCF-7 or OVCAR-3 cells with 323/A3-GUS prior to Epi-glu exposure resulted in an IC50 similar to that for Epi (Figure 5). Even a 4-h drug exposure time was sufficient to completely reverse activity of Epi-glu to Epi (data not shown). Co-incubation with excess cold antibody (100 μg ml$^{-1}$) resulted in an almost complete blockade of binding of the conjugate (2% binding) and a much smaller shift in the reversal of Epi-glu to Epi. For antigen-negative A2780 cells pretreatment with the conjugate had litle effect on Epi-glu cytotoxicity.

### Discussion

Several enzymes conjugated to MAbs are currently being investigated for the selective activation of prodrugs at the tumour site. The present study indicates that β-glucuronidase may be used to activate glucuronidated drugs. We showed that the prodrug Epi-glu is 100–1,000 times less toxic than Epi and can be completely hydrolysed by the GUS-MAb conjugate bound to tumour cells into the active cytotoxic

**Figure 4** Cellular uptake of Epi (O) and Epi-glu (●) at 10 μM by OVCAR-3 cells, as measured by fluorescence after solubilisation of the cells.

**Table I** IC50 of Epi and Epi-glu after 24-h exposure

| | *A2780*[a] | *OVCAR-3*[a] | *MCF-7*[a] |
|---|---|---|---|
| Epi | 0.003 ± 0.001 | 0.2 ± 0.1 | 0.1 ± 0.1 |
| Epi-glu | >20 | >20 | >20 |
| Epi-glu + conj.[b] | 0.5 ± 0.2 | 0.4 ± 0.1 | 0.5 ± 0.3 |
| Epi-glu + conj. + MAb[c] | 0.3 ± 0.2 | 3.0 ± 0.5 | 2.5 ± 1.0 |

[a]IC50 expressed in μM ± s.d., results from three separate experiments. [b]Cells pretreated with 323/A3-GUS conjugate at 5 μg ml$^{-1}$. [c]Cells pretreated with 323/A3-GUS conjugate at 5 μg ml$^{-1}$, with excess 323/A3 at 100 μg ml$^{-1}$.

**Figure 5** Growth inhibition of Epi (O), Epi-glu (●), and Epi-glu after preincubation with 323/A3-GUS conjugate (■). Cells were exposed to the drugs for 24 h. Growth was measured with sulforhodamine B at 72 h.

drug Epi. Even at non-saturating concentrations, probably found in the *in vivo* situation, a remarkable cytotoxic effect could be obtained in antigen-positive tumour cells.

Epi is an active anti-tumour agent in patients with breast cancer, lymphomas, ovarian cancer, and soft-tissue sarcomas (Cersosimo & Ki Hong, 1986). Epi-glu is a naturally occurring metabolite in patients treated with Epi and the pharmacokinetics are well known (Mross *et al.*, 1988). We used Epi-glu as a model prodrug. Our hypothesis that this prodrug would be less toxic than Epi, but upon hydrolysis, would be as active as Epi was confirmed. We demonstrated that the low cytotoxicity of Epi-glu was caused by its decreased cellular uptake. Epi-glu was stable in serum and *in vivo* in BALB/c mice. Epi-glu was not hydrolysed by tumour cells, but complete activation occurred by 323/A3-GUS bound to the tumour cells. These characteristics contrast favourably with other prodrugs used for antibody-directed enzyme targeting. For instance, etoposide phosphate (Senter *et al.*, 1988; Haisma *et al.*, 1992) and p-N-bis (2-chloroethyl) aminobenzoyl glutamic acid derivative (Bagshawe *et al.*, 1988) were less stable and p-di-2-chloroethylaminophenol-β-D-glucuronide (Roffler, 1991) was only 10-times less toxic than the parent compound.

The enzyme selected for conversion of the prodrug into the active drug, should preferably be of human origin to minimise an antibody response in patients. This makes the use of prokaryotic enzymes such as carboxypeptidase (Bagshawe *et al.*, 1988), penicillin-V-amidase (Kerr *et al.*, 1990) and β-lactamase (Shepherd *et al.*, 1991; Alexander *et al.*, 1991) less attractive. Also, the enzyme should be active at a pH found in the extracellular space of tumours, which is acid to near neutrality (Tannock & Rotin, 1989). This will reduce the possibility to explore alkaline phosphatase for prodrug activation. The latter enzyme is also abundantly present in blood and normal tissues, which will lead to the untimely activation of the prodrug. Recently, Haenseler *et al.* (1992) have reported on the use of bovine pancreas carboxypeptidase A to activate the prodrug methotrexate-α-alanine. The prodrug was approximately 150 times less toxic than the drug, but was not completely hydrolysed by the enzyme. This resulted in only a 6-fold difference in cytotoxicity for cells treated with the prodrug alone as compared to treatment with the prodrug combined with the antibody-enzyme conjugate. Unfortunately, no data were presented on the *in vivo* stability of the prodrug or the enzyme.

We used GUS from *E. coli* because it was readily available. The human enzyme may be less efficient, because its pH optimum is 5.4 as compared to 6.8 for *E. coli*-derived GUS. Also, the human enzyme has a lower turnover rate (Dutton, 1966; Fishman, 1970). In fact, the Vmax of 39 nmol $min^{-1}$ $mg^{-1}$ of *E. coli*-derived GUS is low as compared to that for the synthetic substrate p-nitrophenyl-β-D-glucuronide. Therefore, alternative routes for glucuronidation of the Epi molecule or other cytostatic agents should be explored, which could even lead to efficient hydrolysis by GUS from human origin.

The human GUS enzyme should be less immunogenic in patients. For ultimate treatment of cancer based on antibody-enzyme prodrug targeting the group of Bosslet *et al.* (1992) has produced a fusion protein by molecular biology techniques consisting of human GUS and the humanised Fab′ of MAb BW431/26. Studies are in progress to evaluate the efficiency of this conjugate in the hydrolysis of Epi-glu *in vitro* as well *in vivo*.

In our experiments we have demonstrated the potential usefulness of *E. coli*-derived GUS as an enzyme to be used for specific prodrug activation after conjugation to MAbs. Further studies will include the validation of the concept in tumour-bearing animals and will mainly focus on the value of human GUS in this new treatment approach.

### References

ALEXANDER, R., BEELEY, N.R.A., O'DRISCOLL, M., O'NEILL, F.P., MILLICAN, T.A., PRATT, A.J. & WILLENBROCK, F.W. (1991). Celhalosporin nitrogen mustard cabamate prodrugs for 'ADEPT'. *Tetrahedron Lett.*, **32**, 3269–3272.

BAGSHAWE, K.D., SPRINGER, C.J., SEARLE, F., ANTONIW, P., SHARMA, S.K., MELTON, R.G. & SHERWOOD, R.F. (1988). A cytotoxic agent can be generated selectively at cancer sites. *Br. J. Cancer*, **58**, 700–703.

BOSSLET, K., CZECH, J., LORENZ, P., SEDLACEK, H.H., SCHUERMANN, M. & SEEMAN, G. (1992). Molecular and functional characteristics of a fusion protein suited for specific prodrug activation. *Br. J. Cancer*, **65**, 234–238.

CERSOSIMO, R.J. & KI KONG, W. (1986). Epirubicin: a review of the pharmacology, clinical activity, and adverse effects of an adriamycin analogue. *J. Clin. Onc.*, **4**, 425–439.

DUTTON, G.J. (1966). *Glucuronic Acid Free and Combined.* Academic Press: New York, London.

EDWARDS, D.P., GRZYB, K.T., DRESSLER, L.G., MANSEL, R.E., ZARA, D.T., SLEDGE, G.W. & MCGUIRE, W.L. (1986). Monoclonal antibody identification and characterization of a Mr 43,000 membrane glycoprotein associated with human breast cancer. *Cancer Res.*, **46**, 1306–1311.

EVA, A., ROBBINS, K.C., ANDERSEN, D.R., SCINIVASAN, A., TRONICK, S.R., REDDY, E.P., ELLMORE, N.W., GALEN, A.T., LAUTENBERGER, J.A., PAPAS, T.S., WESTIN, E.H., WONG-STAAL, F. & GALLO, R.C., AARONSON, S.A. (1982). Cellular genes analogues to retroviral onc genes are transcribed in human tumour cells. *Science*, **295**, 116–118.

FISHMAN, W.H. (1970). *Metabolic Conjugation and Metabolic Hydrolysis.* Academic Press: New York, London.

HAISMA, H.J., HILGERS, J. & ZURAWSKI, V.R. Jr (1986). Iodination of monoclonal antibodies for diagnosis and radiotherapy using a convenient one vial method. *J. Nucl. Med.*, **27**, 1890–1895.

HAISMA, H.J. (1991). Monoclonal antibodies. In *The Nude Mouse in Oncology Research.* Boven, E. & Winograd, B. (ed.). CRC Press: Boca Raton, Fla, p. 231–246.

HAISMA, H.J., BOVEN, E., VAN MUIJEN, M., DE VRIES, R. & PINEDO, H.M. (1992). Analysis of a conjugate between an anti-carcinoembryonic antigen monoclonal antibody and alkaline phosphatase for specific activation of the prodrug etoposide-phosphate. *Cancer Immunol. Immunother.*, **34**, 343–348.

HAMILTON, T.C., YOUNG, R.C., LOUIE, K.G., BEHRENS, B.C., MCKOY, W.M., GROTZINGER, K.R. & OZOLS, R.F. (1984). Characterization of a xenograft model of human ovarian carcinoma which produces ascites and intraabdominal carcinomatosis in mice. *Cancer Res.*, **44**, 5286–5290.

HAENSELER, E., ESSWEIN, A., VITOLS, K.S., MONTEJANO, Y., MUELLER, B.M., REISFELD, R.A. & HUENNEKENS, F.M. (1992). Activation of methotrexate-α-alanine by carboxypeptidase A-monoclonal antibody conjugate. *Biochemistry*, **31**, 891–897.

KERR, D.A., SENTER, P.D., BURNETT, W.V., HIRSCHBERG, D.L., HELLSTROM, I. & HELLSTROM, K. (1990). Antibody-penicillin-V-amidase conjugates kill antigen positive cells when combined with doxorubicin phenoxyacetamide. *Cancer Immunol. Immunother.*, **31**, 202–205.

LINDMO, T., BOVEN, E., CUTTITTA, F., FEDORKO, J. & BUNN, P.A.J. Jr (1984). Determination of the immunoreactive fraction of radiolabeled monoclonal antibodies by linear extrapolation to binding at infinite antigen excess. *J. Immunol. Methods*, **72**, 77–82.

MAAS, I.W.H.M., BOVEN, E., PINEDO, H.M., SCHLUPER, H.M.M. & HAISMA, H.J. (1991). The effects of γ-interferon combined with 5-fluorouracil or 5-fluoro-2′-deoxyuridine on proliferation and antigen expression in a panel of human colorectal cell lines. *Int. J. Cancer*, **48**, 749–756.

MROSS, K., MAESSEN, P., VAN DER VIJGH, W.J.F., GALL, H., BOVEN, E. & PINEDO, H.M. (1988). Pharmacokinetics and metabolism of epidoxorubicin and doxorubicin in humans. *J. Clin. Onc.*, **6**, 517–526.

ROFFLER, S.R., WANG, S., CHERN, M., YEH, M.Y. & TUNG, E. (1991). Anti-neoplastic glucuronide prodrug treatment of human tumor cells targeted with a monoclonal antibody-enzyme conjugate. *Biochem. Pharmacol.*, **42**, 2062–2065.

SENTER, P.D., SAULNIER, M.G., SCHREIBER, G.J., HIRSCHBERG, D.L., BROWN, J.P., HELLSTROM, I. & HELLSTROM, K.E. (1988). Anti-tumor effects of antibody-alkaline phosphatase conjugates in combination with etoposide phosphate. *Proc. Natl Acad. Sci. USA*, **85**, 4842–4845.

SHEPHERD, T.A., JUNGHEIM, L.N., MEYER, D.L. & STARLING, J. (1991). A novel targeted delivery system using a cephalosporin-oncolytic prodrug activated by an antibody-β-lactamase conjugate for the treatment of cancer. *Bioorg. Med. Chem. Lett.*, **1**, 21–26.

SOULE, H.D., VAZQUEZ, J., LONG, A., ALBERT, S. & BRENNAN, M. (1973). A human cell line from a pleural effusion derived from a breast carcinoma. *J. Natl Cancer Inst.*, **51**, 1409–1415.

TANNOCK, I.F. & ROTIN, D. (1989). Acid pH in tumors and its potential for therapeutic exploitation. *Cancer Res.*, **49**, 4373–4384.